# Validation of a Parent-Reported Physical Activity Questionnaire by Accelerometry in European Children Aged from 6 to 12 Years Old

**DOI:** 10.3390/ijerph19159178

**Published:** 2022-07-27

**Authors:** Daniel Prieto-Botella, Desirée Valera-Gran, Loreto Santa-Marina, Izaro Babarro, Mikel Subiza-Pérez, Maribel Casas, Mónica Guxens, Gabriela Cárdenas-Fuentes, Barbara Heude, Jonathan Y. Bernard, Rosemary R. C. McEachan, Judith García-Aymerich, Martine Vrijheid, Eva-María Navarrete-Muñoz

**Affiliations:** 1 Department of Surgery and Pathology, Miguel Hernandez University, 03550 Alicante, Spain; dprieto@umh.es (D.P.-B.); enavarrete@umh.es (E.-M.N.-M.); 2 Grupo de Investigación en Terapia Ocupacional (InTeO), Miguel Hernández University, 03550 Alicante, Spain; 3 Spanish Consortium for Research on Epidemiology and Public Health (CIBERESP), 28029 Madrid, Spain; ambien4ss-san@euskadi.eus (L.S.-M.); mikel.subiza@ehu.eus (M.S.-P.); maribel.casas@isglobal.org (M.C.); monica.guxens@isglobal.org (M.G.); judith.garcia@isglobal.org (J.G.-A.); martine.vrijheid@isglobal.org (M.V.); 4 Group of Environmental Epidemiology and Child Development, Biodonostia Health Research Institute, 20014 San Sebastian, Spain; izaro.babarro@ehu.eus; 5 Ministry of Health of the Basque Government, SubDirectorate for Public Health and Addictions of Gipuzkoa, 20010 San Sebastián, Spain; 6 Department of Clinical and Health Psychology and Research Methods, University of the Basque Country UPV/EHU, 20018 San Sebastián, Spain; 7 ISGlobal, 08036 Barcelona, Spain; gabriela.cardenas@isglobal.org; 8 Departament de Ciències Experimentals i de la Salut, Universitat Pompeu Fabra (UPF), 08002 Barcelona, Spain; 9 Department of Child and Adolescent Psychiatry/Psychology, Erasmus University Medical Centre-Sophia Children’s Hospital, 3015 Rotterdam, The Netherlands; 10 Centre for Research in Epidemiology and StatisticS (CRESS), Université Paris Cité, Inserm, INRAE, F-75004 Paris, France; barbara.heude@inserm.fr (B.H.); jonathan.bernard@inserm.fr (J.Y.B.); 11 Bradford Institute for Health Research, Bradford BD9 6RJ, UK; rosie.mceachan@bthft.nhs.uk; 12 Alicante Institute for Health and Biomedical Research, ISABIAL-UMH, 03010 Alicante, Spain

**Keywords:** moderate-to-vigorous physical activity, validity measures, measurement, childhood

## Abstract

Validated physical activity (PA) questionnaires are crucial for collecting information in large epidemiological studies during childhood. Thus, this study analyzed the validity of a parent-reported PA questionnaire based on the Children’s Leisure Activities Study Survey by accelerometry in European children aged from 6 to 12 years old. We used data from 230 children of the Human Early-Life Exposome and Infancia y Medio Ambiente projects. Mean differences between moderate-to-vigorous PA (MVPA) reported by the questionnaire and the accelerometer were calculated (min/day), and its associated factors were explored by multiple robust linear regression. The agreement between methods was examined using a Bland–Altman plot. The concurrent validity of assessing MVPA was analyzed by cohort-adjusted Spearman’s partial correlations. ROC curve analysis was also used to explore the questionnaire’s capability to identify active children based on the World Health Organization guidelines. A moderate correlation was found between parent-reported and accelerometer MVPA (rho = 0.41, *p* < 0.001). The child’s sex (girl) was statistically associated with the mean MVPA difference between methods. However, this questionnaire accurately identified physically active children (area under the curve = 83.8% and 82.7% for boys and girls, cut-points = 68.6 and 45.4 min/day in MVPA, respectively). Consequently, this questionnaire is suitable for classifying active children in order to monitor public health interventions regarding PA.

## 1. Introduction

Recently, the benefits of physical activity (PA) on school-aged children’s physical and mental health have been well-documented [1,2,3,4]. Specifically, PA has been associated with an improvement in cardiometabolic health, musculoskeletal fitness, bone development, and cognitive outcomes, helping to reduce adiposity and enhancing children’s psychological well-being [1,2,3,4,5]. However, these positive findings seem to depend on the dose and intensity of daily PA [5,6]. For these reasons, it is crucial to use reliable and valid instruments to monitor PA in childhood.

In large epidemiological studies, self-reported or parent-reported questionnaires have been suggested as good options to assess PA in children, mainly because they are low-cost and easy-to-administer instruments for collecting a vast amount of information [7]. However, one of the main disadvantages of self-reported questionnaires is that they are based on memory recall, thereby involving potential information biases [8,9,10]. Therefore, to control the variability of these subjective methods, it is essential to validate these questionnaires by comparing them with objective methods with different measurement errors (e.g., accelerometers or pedometers). A validation study can provide information on the structure and magnitude of the measurement error, and it can help in adjusting subsequent analysis and choosing a suitable statistical approach [11].

According to a recently updated systematic review of childhood PA assessment tools, there are a large number of validated questionnaires for measuring PA in children and adolescents [12]. This systematic review reported that 29 out of the 89 evaluated questionnaires were validated in children aged between 6 and 12 years old [12]. Nine of these PA questionnaires, such as the Physical Activity Questionnaire for Older Children (PAQ-C) and the Patient Assessment and Council for Exercise (PACE) [13,14], were validated in European children. However, only the PAQ-C and the ENERGY questionnaires were validated across several European countries [15]. These two PA questionnaires were created to assess general PA levels in children, but they did not provide specific daily frequency, time, or intensity estimates. The benefits of PA are related to the daily dose and intensity [5,6]; therefore, validating PA questionnaires including these parameters for the use in school-aged children remains an issue for large epidemiological studies. 

Thus, this study aimed to explore the validity of a parent-reported PA questionnaire based on the Children’s Leisure Activities Study Survey (CLASS) [16] by accelerometry in European children aged 6 to 12 years old.

## 2. Materials and Methods

### 2.1. Study Population

This study included 230 children with complete information on PA measured by questionnaire and accelerometer from different European population-based birth cohorts, the Human Early Life Exposome (HELIX, http://www.projecthelix.eu/index.php/en), and Infancia y Medio Ambiente (INMA, www.proyectoinma.org) projects. Detailed information on study populations and epidemiological designs of both projects has been published elsewhere [17,18]. The HELIX study is a collaborative project including the HELIX Child Panel; a study developed between 2013 and 2016 with children aged from 6 to 11 years [19]. In the present study, we used data available on child’s PA from three population-based birth cohorts included in the HELIX Child Panel: BiB (Born in Bradford, United Kingdom, n = 27), EDEN (Étude des Déterminants pré et postnatals du développement et de la santé de l’Enfant, France, n = 30), and INMA-Sabadell (Spain, n = 37). The children’s mean (standard deviation, sd) age in these cohorts was 6.7 (0.2), 10.9 (0.4), and 8.6 (0.6) years, respectively. Regarding the INMA project, we used complete data on PA of 136 children aged between 10 and 12 years old (mean = 10.7, sd = 0.2) from the follow-up performed between 2017 and 2020 in the INMA-Gipuzkoa cohort. All participants provided signed informed consent, and the respective ethical committees granted ethical approval for each study.

### 2.2. Questionnaire

PA was assessed by a parent-reported questionnaire based on the CLASS [16]. This questionnaire included PA assessments during school time and outside of school. Each of these parts contains a list of different activities (e.g., running, tennis, or basketball) plus the possibility to add an unspecified activity. Parents were asked: “During a typical week, does your child do any further programmed physical activities during school time?” and “Thinking about the rest of the year: does your child do any further physical activities (including planned activity or just normal play) each day outside of school hours?” followed by a question about the daily frequency and duration in minutes of the selected activity. A metabolic equivalent of task (MET) value was assigned to every activity based on previous publications on childhood and youth [20,21]. Mean minutes per day in moderate-to-vigorous PA (MVPA, ≥3 METs) were calculated as the sum of the minutes per day in MVPA divided by seven.

### 2.3. Accelerometer

PA was assessed using an ActiGraph wGT3X-BT tri-axial accelerometer (Pensacola, FL, USA) in the Helix Child Panel study, and an Activinsights GeneActiv tri-axial accelerometer (Kimbolton, UK) in the INMA-Gipuzkoa, which were worn during the same week the questionnaire was administered. Data reduction regarding both accelerometers was conducted with similar methods.

ActiGraph records raw acceleration data at a rate between 30 and 100 Hz with a memory capacity of up to 4 GB. In addition, it is characterized by being light (19 g) and small (4.6 × 3.3 × 1.5 cm). The accelerometer was worn on the right side of the hip for one week, only during the day. Raw accelerometer data were collected at 30 Hz. After data collection, ActiGraph data were downloaded and converted into 10 s epochs for post-processing using ActiLife software. The METs of each epoch were calculated using the vertical axis method, in accordance with Crouter et al. [22]. A cut-point of ≥3 METs was established for MVPA [22]. Non-wear time was defined as 20 min or more of consecutive zero counts, and waking hours were set between 6:00 and 23:59 [23,24]. In addition, a minimum of 10 h/day and at least three days/week of accelerometer data were needed for inclusion in the analysis [25]. Mean MVPA minutes per day were calculated for each child as follows: sum of the accelerometer minutes/number of valid days [26].

Similarly, the GeneActiv accelerometer is characterized as being light (16 g) and small (3.6 × 3.0 × 1.2 cm). It records raw acceleration data at a rate of up to 100 Hz. The accelerometer was worn on the non-dominant wrist for seven days in a 24 h regime, and data were collected at 85.7 Hz. After data collection, raw data were downloaded without time spans in CSV files using GENEActiv software. Raw data post-processing was conducted with the GGIR R package version 2.4 (https://github.com/wadpac/GGIR, accessed on 14 April 2022), which has been designed for processing raw accelerometer data [27]. Daily PA intensities were generated daily using the milli g cut-points published by Hildebrand et al.: sedentary–light = 56.3 mg; light–moderate = 191.6 mg; and moderate–vigorous = 695.8 mg [28,29]. These cut-points were developed to be equivalent to MET intensities. Non-wear time was identified as 15 min blocks based on the characteristics of the 60 min window, as described in previous publications [30]. Finally, waking hours, valid data, and summary variables were established based on the same criteria used for ActiGraph.

### 2.4. Study Covariates

Covariates included child sociodemographic and anthropometric data: cohort, age (years), sex, body mass index (BMI, kg/m^2^), and BMI groups (overweight/obese, normal weight) based on the age- and sex-specific World Health Organization (WHO) cut-points [31]. In addition, the mother’s age at delivery (years) and family’s socioeconomic status (low, middle, and high) were also collected.

### 2.5. Statistical Analysis

R software version 4.1.2 (R Core Team, R Foundation for Statistical Computing, Vienna, Austria; http://www.R-project.org) was used to perform the statistical analysis. All statistical tests were bilateral, with an applied significance level of 0.05. The Kolmogorov–Smirnov test with Lilliefors correction was used to check the normality of continuous variables.

First, descriptive analyses of the mother–child pairs’ sociodemographic and anthropometric characteristics were conducted using frequencies and percentages. Bivariate chi-squared tests were performed to evaluate differences between cohorts. Second, the mean differences between parent-reported and accelerometer MVPA (min/day) measures were calculated and presented in a Bland–Altman plot [32]. The possible covariates associated with this difference were explored by performing a multiple robust linear regression through the “robustbase” R package [33]. Third, the concurrent validity between parent-reported and accelerometer MVPA measures was evaluated by a cohort-adjusted partial Spearman’s correlation (“ppcor” R package) [34]. Several sensitivity analyses were conducted to examine the main findings’ robustness by separately adjusting the main correlation model by the child (sex, age, and BMI) and mother (age and socioeconomic status) characteristics. Mother’s age was categorized into <31 years and ≥31 years based on the median value. Before performing the validity analysis, heterogeneity among the study cohorts was evaluated. The cohort-separated correlations were analyzed using meta-analytic techniques, and the heterogeneity was quantified using I^2^ statistics with the “metacor” R package [35,36]. The obtained I^2^ value was <25%.

Finally, we explored the capability of the questionnaire to classify children as “physically active” based on the WHO guidelines (at least 60 min/day of MVPA) [5] through the analysis of the receiver operating characteristic (ROC) curve, stratifying by children’s sex (“pROC” R package) [37]. In addition, we calculated the sensitivity, specificity, positive predictive value, and negative predictive value using the optimal questionnaire threshold identified by ROC curve analysis.

## 3. Results

### 3.1. Characteristics of the Study Population

Table 1 describes the general characteristics of the mother–child pairs by cohort. On average, 52.2% of the children were girls, ranging from 37.8% to 58.2% through cohorts. In addition, BMI groups were similarly distributed among cohorts (*p* = 0.17), with an overweight/obese total percentage of 33.5%. Regarding the maternal age at delivery, 128 (55.7%) mothers were aged ≥ 31 years. The youngest mothers were found in BiB and EDEN (66.7%, respectively). The majority of the families’ socioeconomic situation was low/middle (60.0%). However, an over-representation of families with a high socioeconomic status was observed in EDEN (80.0%).

### 3.2. Mean MVPA Levels

As measured with the accelerometer, children spent an average (standard deviation) of 72.8 (43.2) min/day in MVPA. In comparison, the minutes/day in MVPA collected through questionnaire were slightly higher, being 79.2 (58.0) (Table 2). The child’s sex was associated with the difference recorded between the two methods (β girls vs. boys = 15.2, 95%CI = 2.3 to 28.1, *p* = 0.020). On average, parents whose children were girls over-reported 11.9 min/day of MVPA. No other associations were observed (Table 2).

### 3.3. Concurrent Validity

Spearman’s partial correlation adjusted for cohort between parent-reported and accelerometer MVPA time was 0.41 (95%CI = 0.30 to 0.51, *p* < 0.001). This correlation remained almost of the same strength after adjusting for other relevant variables such as the child’s age (rho = 0.41, 95%CI = 0.30 to 0.51, *p* < 0.001) or BMI (rho = 0.40, 95%CI = 0.29 to 0.51, *p* < 0.001). A slight decrease was only observed when adjusting for the child’s sex (rho = 0.38, 95%CI = 0.26 to 0.48, *p* < 0.001).

### 3.4. Agreement between Methods

**Figure 1:**
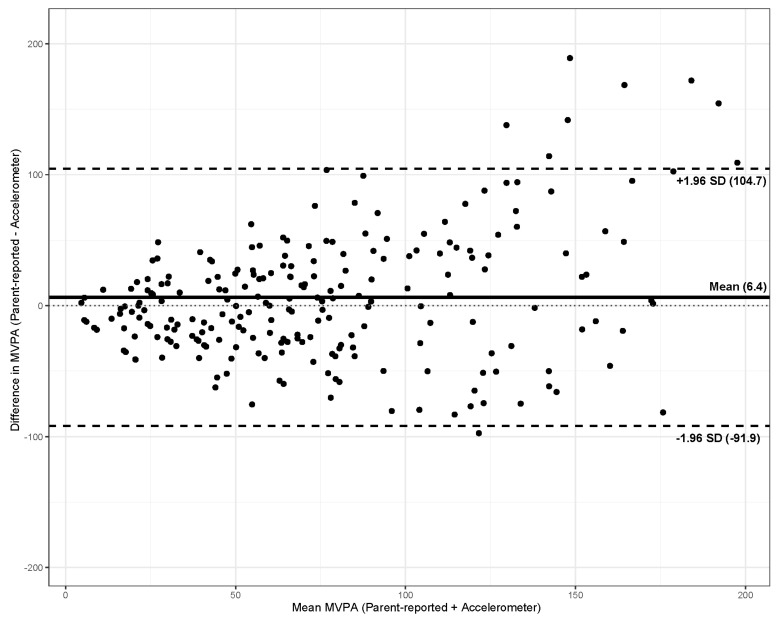
Bland–Altman plot with mean bias (solid line) and 95% limits of agreement (dashed lines) between the children’s parent-reported and accelerometer measurements of moderate-to-vigorous physical activity (MVPA, min/day).

The Bland–Altman plot (Figure 1) showed a mean bias between methods (parent-reported vs. accelerometer) of 6.4 min/day of MVPA (95% limits of agreement = −91.9 to 104.7). A total of nine cases (3.9%) were outside the limits of agreement. Children with a mean MVPA (i.e., parent-reported + accelerometer) > 100 min/day tended to present higher differences between methods.

### 3.5. Known-Groups Validity

There were 51.7% of girls and 60.0% of boys classified as physically active (≥60 min/day of MVPA) based on the accelerometer. The ROC curve analysis showed an area under the curve (AUC) of 83.8% (95%CI = 76.6 to 91.0%) and 82.7% (95%CI = 75.2 to 90.3%) for girls and boys, respectively (Table 3). In addition, this analysis established an optimal threshold of 68.6 min/day of MVPA for classifying active boys based on the parent-reported questionnaire (sensitivity = 72.7%, specificity = 79.5%). However, this optimal threshold was lower in girls (45.4 min/day of MVPA, sensitivity = 96.8, specificity = 60.3).

## 4. Discussion

The results suggested a moderate correlation between parent-reported and accelerometer MVPA. Although the Bland–Altman plot showed an acceptable agreement across methods, the error and overestimation tended to rise as mean MVPA increased. Specifically, children with a mean MVPA (i.e., parent-reported + accelerometer) > 100 min/day showed greater differences between methods. Interestingly, this difference was associated with the child’s sex, with parents of girls being more prone to overestimate their daughter’s MVPA. The optimal questionnaire MVPA threshold identified by the ROC curve analysis was lower in girls in comparison with boys. However, this assessment tool showed an adequate accuracy in identifying physically active children.

The correlation between parent-reported and accelerometry MVPA found in this study was moderate (rho = 0.41), but in line with the current literature. A recent review published by Hidding and colleagues found that barely 7 out of 29 child-oriented (≥6 to  <12 years) PA questionnaires had correlation values above 0.50 [12]. A possible explanation for these moderate correlations could be that children may not accurately recall the amount of time spent in PA due to cognitive immaturity and variable activity patterns, which are more challenging to remember [38,39]. However, in our case, parent reporting can, to some extent, vary from direct measurements because parents might have difficulty adequately quantifying the PA of their children, taking into account the activities performed in their spare time and those at the school [38]. Furthermore, it should be noted that PA measured by the questionnaire may differ from that measured by an accelerometer. The questionnaire asks about PA during a “typical week,” whereas the accelerometers recorded data from a specific week, suggesting that PA could be different.

Our findings also showed that parents tended to slightly overestimate (6.4 min/day on average) the levels of MVPA of their children. The literature on validation studies indicates that self-reported methods tend to over-report PA. According to the review conducted by Adamo and colleagues, 72% of the reviewed child-oriented indirect methods, including questionnaires, overestimated the PA values measured by direct methods, showing differences between methods ranging from −95% to 13,025% [40]. Similar results can be observed in the review by Hidding and colleagues, where MVPA mean differences ranged from −15.6 to 117.6 min [12]. Nevertheless, our results were among the estimates with the lowest differences between a PA questionnaire vs. accelerometry found by Hidding or Adamo [12,40]. The potential recall and information biases previously identified could explain this difference [38,39]. In the present study, the difference between methods was associated with the child’s sex, because girls’ parents tended to overestimate their daughter’s activity. A possible explanation could rely on the observed lower PA level of girls compared with boys, which has been evidenced in previous studies [41,42]. It has been theorized that parents may compare their child with other children of the same age and sex when judging and compiling their child’s PA level [43]. Therefore, girls’ parents could be more prone to overestimate their daughter’s PA due to their frame of reference [43]. Consistent with other studies, this result highlights the importance of taking sex into account in PA research during the early stages of life [43,44,45].

The Bland–Altman plot analysis showed a good agreement between methods, suggesting that the analyzed questionnaire provided reasonable estimates of daily MVPA. However, a slight positive magnitude bias was observed, in line with other published validation studies [12]. Children with a mean MVPA (i.e., parent-reported + accelerometer) > 100 min/day presented greater differences between methods. The Chinese version of the CLASS also observed this bias in a similar group of participants [46]. The authors suggested that this bias could be attributable to the intermittent nature of PA, because children usually do not engage in sustained PA, but perform it in several bouts. Thus, parents can report that their children play one hour of basketball as MVPA, even though some of the time was possibly less strenuous [46]. Furthermore, the accelerometer protocols establish the removal of the device for contact and water-based sports, leading to a possible under-recording of these activities.

Our questionnaire also showed a good discriminant power for classifying physically active boys and girls based on the WHO guidelines (AUC > 0.8) [5]. Furthermore, the girls’ optimal questionnaire threshold identified by the ROC curve analysis suggested that the questionnaire classified girls as physically active with less MVPA time (45.4 min/day) than the accelerometer (≥60 min/day) in comparison with boys (68.6 min/day). In light of our findings, this could be explained by the lower MVPA of girls vs. boys, which is in line with previous studies in children [41,42]. Although the underlying reasons still need to be clarified, it has been theorized that, due to the girls’ lower PA level, parents may expect their daughters’ playing style to be quieter and to spend less time engaging in sports [47].

In this study, several limitations should be considered before interpretation of the findings. First, the analyzed sample may not be representative of the total population because it was part of a subsample of HELIX and INMA projects. However, this study had a multi-centric European design that could provide valuable insights into the involved cohorts. Second, our sample could be over-represented by the Gipuzkoa participants, who were also evaluated with a different accelerometer compared with the other cohorts. However, we observed a similar MVPA correlation value after excluding the children from Gipuzkoa (data not shown), performing accelerometer data reduction using shared criteria to address this limitation. Finally, we considered the child’s sex in our analysis to account for possible measurement differences, and the validity of the results was first explored using meta-analytic techniques to identify a possible heterogeneity between cohorts.

## 5. Conclusions

The validity of assessing MVPA level between the parent-reported questionnaire and the accelerometer was moderate. This questionnaire could be suitable for identifying physically active children, although the estimates for PA duration should be treated with caution. In addition, we would like to emphasize the importance of considering the child’s sex when evaluating or using PA data. Nevertheless, because accelerometry devices are costly, increase participant burden, and do not provide information on activity type, the use of this questionnaire can be appropriate for monitoring public health interventions regarding PA.

## Figures and Tables

**Table 1:** Characteristics of mother-child pairs according to cohort.

Child’s Characteristics, n (%)	Total (n = 230)	Children Aged 6–11			Children Aged 10–12	*p* ^a^
		BiB (n = 27)	INMA-Sabadell (n = 37)	EDEN (n = 30)	INMA-Gipuzkoa (n = 136)	
Sex						0.064
Boys	110 (47.8)	13 (48.1)	23 (62.2)	18 (60.0)	56 (41.2)	
Girls	120 (52.2)	14 (51.9)	14 (37.8)	12 (40.0)	80 (58.2)	
BMI groups ^b^						0.174
Overweight/obese	77 (33.5)	8 (29.6)	14 (37.8)	5 (16.6)	50 (36.8)	
Normal weight	153 (66.5)	19 (70.4)	23 (62.2)	25 (83.4)	86 (63.2)	
**Mother’s characteristics, n (%)**						
Age groups ^c^						<0.001
<31 years	102 (44.3)	18 (66.7)	12 (32.4)	20 (66.7)	52 (38.2)	
≥31 years	128 (55.7)	9 (33.3)	25 (67.6)	10 (33.3)	84 (61.8)	
Socioeconomic status						<0.001
High	92 (40.0)	9 (33.3)	14 (37.8)	24 (80.0)	45 (33.1)	
Low/Middle	138 (60.0)	18 (66.7)	23 (62.2)	6 (20.0)	91 (66.9)	

^a^ Chi-squared tests across cohorts. ^b^ C Age- and sex-specific World Health Organization cut-points. ^c^ Cut-point based on the median value.

**Table 2:** Linear regression of the difference between questionnaire and accelerometer MVPA time adjusted for child’s and mother’s characteristics.

	n	MVPA (Min/Day)				
		Questionnaire	Accelerometer	Questionnaire–Accelerometer		
		Mean (SD)	Mean (SD)	Difference	β ^a^ (95%CI)	*p*
**Total**	230	79.2 (58.0)	72.8 (43.2)	6.4	-	-
Cohort						0.781
BiB	27	52.3 (43.2)	42.1 (19.5)	10.2	Ref	
INMA-Sabadell	37	43.4 (27.0)	49.8 (24.3)	−6.4	−8.9 (−31.2 to 13.5)	
EDEN	30	26.0 (22.9)	31.1 (21.8)	−5.1	−8.7 (−28.1 to 10.8)	
INMA-Gipuzkoa	136	106.0 (56.4)	94.4 (40.5)	11.6	−4.0 (−25.4 to 17.3)	
**Child’s characteristics**						
Sex						0.020
Boys	110	84.8 (65.0)	84.5 (48.9)	0.3	Ref	
Girls	120	74.1 (50.4)	62.2 (34.1)	11.9	15.2 (2.3 to 28.1)	
Age groups ^b^						0.817
≤10.6 years	114	61.3 (46.2)	58.2 (33.2)	2.8	Ref	
>10.6 years	116	96.8 (63.0)	87.2 (47.0)	9.6	1.9 (−14.4 to 18.2)	
BMI ^c^						0.299
Overweight/obese	77	79.4 (58.8)	69.0 (43.7)	10.4	Ref	
Normal	153	79.1 (57.8)	74.7 (42.9)	4.4	−6.8 (−19.6 to 6.1)	
**Mother’s characteristics**						
Age groups ^b^						0.451
<31 years	102	73.5 (55.5)	68.4 (44.6)	5.1	Ref	
≥31 years	128	83.8 (59.7)	76.4 (41.8)	7.4	−4.6 (−16.5 to 7.4)	
Socioeconomic status						0.856
High	92	66.7 (51.4)	64.5 (44.4)	2.2	Ref	
Low/Middle	138	87.6 (60.8)	78.4 (41.6)	9.2	1.1 (−11.1 to 13.3)	

Abbreviations: MVPA, moderate-to-vigorous physical activity; SD, standard deviation; CI, confidence interval. ^a^ Robust linear regression β coefficients adjusted for cohort and all child’s and mother’s characteristics. ^b^ Cut-point based on the median value. ^c^ Age- and sex-specific World Health Organization cut-points.

**Table 3:** Validity to identify children who accumulated ≥ 60 versus <60 min/day of MVPA based on the accelerometry using the parent-reported questionnaire optimal thresholds.

	Girls (n = 120)	Boys (n = 110)
Parent-reported MVPA threshold (min/day) ^a^	45.4	68.6
Sensitivity (95%CI)	96.8% (88.8 to 99.6)	72.7% (60.4 to 83.0)
Specificity (95%CI)	60.3% (46.6 to 73.0)	79.5% (64.7 to 90.2)
Positive predictive value (95%CI)	72.3% (61.4 to 81.6)	84.2% (72.1 to 92.5)
Negative predictive value (95%CI)	94.6% (81.8 to 99.3)	66.0% (51.7 to 78.5)
Area under the curve (95%CI) ^a^	83.8% (76.6 to 91.0)	82.7% (75.2 to 90.3)
Physically active prevalence ^b^	51.7% (42.4 to 60.9)	60.0% (50.2 to 69.2)

Abbreviations: MVPA, moderate-to-vigorous physical activity; CI, confidence interval. ^a^ Based on the receiver operating characteristic (ROC) analysis. ^b^ ≥60 min/day versus <60 min/day of MVPA based on accelerometry.

## Data Availability

Not applicable.
